# Re-emergence of bovine piroplasmosis in Hungary: has the etiological role of *Babesia divergens* been taken over by *B. major* and *Theileria buffeli*?

**DOI:** 10.1186/1756-3305-7-434

**Published:** 2014-09-11

**Authors:** Sándor Hornok, Anita Mester, Nóra Takács, Isabel G Fernández de Mera, José de la Fuente, Róbert Farkas

**Affiliations:** Department of Parasitology and Zoology, Faculty of Veterinary Science, Szent István University, Budapest, Hungary; Veterinary Clinic, Mester tanya, Bátonyterenye, Hungary; SaBio. Instituto de Investigación en Recursos Cinegéticos IREC, CSIC-UCLM-JCCM, Ronda de Toledo s/n, 13005 Ciudad Real, Spain; Department of Veterinary Pathobiology, Center for Veterinary Health Sciences, Oklahoma State University, Stillwater, OK 74078 USA

**Keywords:** Cattle, Babesiosis, Theileriosis, *Anaplasma marginale*, *Anaplasma ovis*, *A. phagocytophilum*

## Abstract

**Background:**

The prevalence of bovine babesiosis caused by *Babesia divergens* has been declining during the past decades in northeastern Hungary, and no cases have been observed since 2008. Infections of cattle with *B. major* and *Theileria buffeli* were hitherto reported in southern and western Europe. In other parts of the globe, there is evidence of emergence and a growing clinical importance of *T. buffeli* and closely related genotypes of the *T. orientalis* complex.

**Findings:**

In a herd of 88 beef cattle kept in northeastern Hungary, bovine piroplasmosis was diagnosed in nine animals through the examination of blood smears or by molecular methods. *B. major* was identified in five animals, two of which died. In addition, four cattle harboured *T. buffeli*, and one of these animals was anaemic. Despite their presence, a contributory role of *Anaplasma marginale* and *A. phagocytophilum* could not be established in the disease cases.

**Conclusions:**

In this study *B. major* and bovine theileriosis is reported for the first time in central-eastern Europe, where clinical cases were associated with a mild winter.

## Findings

### Background

Bovine piroplasmosis is caused by *Babesia* and *Theileria* spp. which are transmitted by ixodid ticks as biological vectors [1, 2]. The geographical ranges of babesioses and theilerioses are defined by the region where competent vectors are indigenous.

Thus, *Babesia divergens*, which is zoonotic and regarded as highly pathogenic to cattle, is widespread in Europe, owing to its vector tick species *Ixodes ricinus* [1]. However, despite its clinical importance, this piroplasm has lost its significance over time in some countries. In northeastern Hungary, the last case of known clinical *B. divergens* infection was diagnosed in May 2008 (GenBank: HQ395757, unpublished observations). Prior to that, during the past decades, a gradual decline was noted in the number of disease cases caused by this piroplasm until it became virtually extinct [3]. This phenomenon, i.e., the decreasing prevalence of clinical *B. divergens* infections, was also shown in Ireland [4] and Norway [5].

On the other hand, *B. major* and *Theileria buffeli*, which are usually considered benign agents, have been reported from a small number of countries in Europe, including the UK, the Netherlands, France, Germany and Spain for *B. major* [6–9] and the UK, Portugal, Spain, Italy and Greece for *T. buffeli* [9–13]. Altogether, countries endemic for both piroplasms appear to be confined to southern and western Europe, corresponding to the regions where their common vector, *Haemaphysalis punctata* occurs [14].

While *B. major* will mostly cause either mild disease or no clinical signs, this species has also been implicated in cases of bovine anaemia (with haematocrit values decreasing to 17%) and haemoglobinuria [6, 9]. Similarly, *T. buffeli* may cause anaemia, oedema, icterus and recumbency in cattle [10, 15, 16]. During the past few years there has been growing concern about the increased involvement of *T. buffeli* and other genotypes of the *T. orientalis* complex in clinical cases, implying the emergence of bovine theileriosis on other continents [16, 17].

Here we report accumulated cases of mild to fatal piroplasmosis in a beef cattle herd in northeastern Hungary with unusual seasonality and causative agents (*B. major*, *T. buffeli*) that are new in the central-eastern European region. Because bovine anaplasmosis could have been important in the differential diagnoses (*Anaplasma marginale* and *A. ovis* occur in the same county in Hungary [18], and *A. phagocytophilum* is similarly known to cause anaemia [19]), the presence of these *Anaplasma* spp. was also evaluated in the herd.

### Methods

The first cattle, which had been grazing the same pastures in northeastern Hungary (Nógrád county, Mátranovák, geographical coordinates: 48° 2' 12.4" N, 19° 58' 35.1" E) in 2012–2013, exhibited clinical signs of piroplasmosis in November, 2013. In the same month, a herd of 88 Charolais beef cattle were transferred from southeastern to northeastern Hungary, to the above locality. In the latter herd, clinical signs of piroplasmosis were noted in two animals within 1.5 months of their arrival, in December, 2013. Relevant animals were kept on pasture in the same area throughout the year.

After the appearance of clinical signs, EDTA blood samples were collected from the coccygeal vein of the three severely affected animals, and the presence of piroplasms was evaluated in Giemsa-stained blood smears. In one animal, complete haematological and biochemical analyses were also performed (Hemo-Vet, Veterinary Clinical Laboratory, Budapest, Hungary). EDTA blood samples were collected from all 85 cattle in the herd in January, 2014. In addition, haematological analysis was performed in blood samples in which piroplasms were identified by molecular methods.

DNA was extracted with the QIAamp Mini Kit (QIAGEN, Hilden, Germany) individually from 200 μl of 85 blood samples, as reported previously [20]. Subsequently, all PCRs were run with the appropriate positive and negative controls. All blood DNA samples were screened for the presence of piroplasms by conventional PCR [21]. In brief, the primers BJ1 (forward: 5'-GTC TTG TAA TTG GAA TGA TGG-3') and BN2 (reverse: 5'-TAG TTT ATG GTT AGG ACT ACG-3') were used to amplify an approximately 500 bp portion of the *18S rRNA* gene of *Babesia*/*Theileria* spp. The reaction volume was 25 μl, i.e., 5 μl of extracted DNA was added to 20 μl of a reaction mixture containing 0.5 U HotStarTaq DNA Plus polymerase (5U/ μl), 200 μM of PCR nucleotide mix, 1 μM of each primer and 2.5 μl of 10× Coral Load PCR buffer (15 mM MgCl_2_ included). Cycling conditions included an initial denaturation step at 95°C for 10 min, followed by 40 cycles of denaturation at 95°C for 30 s, annealing at 54°C for 30 s and extension at 72°C for 40 s. The final extension was performed at 72°C for 5 min. Purification and sequencing of PCR products was performed by Biomi Inc. (Gödöllő, Hungary). Representative sequences were submitted to GenBank (KJ756504 for *B. major*, KJ756505 for *T. buffeli*).

In addition, all cattle blood DNA samples were evaluated for the presence of *Anaplasma* spp*. Anaplasma phagocytophilum* was investigated with a TaqMan real-time PCR that amplifies part of the major surface protein 2 (*msp2*) gene (as described in [20]). Analysis for *A. marginale* was carried out in two steps: first by screening for Anaplasmataceae members by a *16S rRNA* gene conventional PCR (as described in [18]), followed by a conventional PCR for the *msp4* gene of *A. marginale/A. ovis* according to [22]. Sequencing of the cloned product of the latter test was attempted from all animals that were *Babesia* or *Theileria* infected. Obtained sequences were submitted to GenBank (KJ883270-71).

Confidence intervals (CI) for the prevalence rates were calculated at the level of 95%.

### Results

In November and December, three cattle (No. 1–3) showed clinical signs of babesiosis (Table 1), and *B. major* was detected in their blood smears. No *Theileria* or *Anaplasma* inclusion bodies were seen in the red blood cells. Clinical laboratory findings from animal No. 1 (Table 1) were suggestive of pathological changes in parenchymal organs, muscle damage and chronic haemolysis. Despite treatment with 1 ml Imizol® (Intervet International B.V., Boxmeer, the Netherlands) per 100 kg body weight subcutaneously, two of the severely affected cattle died (Table 1).

**Table 1:** **Summary of clinical and laboratory data for cattle infected with piroplasms and** ***Anaplasma marginale*** **/** ***A. ovis***

Animal no.	Month when diseased (tested)	Age in year	Main clinical signs	Laboratory findings			Status/outcome
				Haematocrit	Blood smear	Molecular	
1.	November	3	lethargy, ataxia, oedema, recumbency^1^	27%	Bm	ne	death in 2 wk
2.	December	3	lethargy, oedema, recumbency	ne	Bm	ne	slaughtered
3.	December	10	haemoglobinuria, recumbency	ne	Bm	ne	death in 4 wk
4.	(January)	0.3	-	30%	ne	Bm	healthy
5.	(January)	0.5	-	normal	ne	Bm + Am	healthy
6.	(January)	8	anaemia	22%	ne	Tb	treated: recovery
7-9.	(January)	5,7,10	-	normal	ne	Tb + Am	healthy

^1^Clinical laboratory findings of animal No 1. [normal range]:

- elevated: aspartate-aminotransferase (AST): 337.2 [10–160] IU/l, alkaline phosphatase (AP): 310 [40–200] IU/l, glutamate dehydrogenase (GLDH): 116.8 [10–25] U/l, carbamid: 6.6 [1–3.7] mmol/l, lactate dehydrogenase (LDH): 10050 [<450] U/l.

- lowered: albumin: 23.7 g/l [35–42].

*Abbreviations: Bm* *Babesia major*, *Tb* *Theileria buffeli*, *Am* *Anaplasma marginale/A. ovis*, *ne* not evaluated, *wk* week(s).

In January, PCR positivity to piroplasms was demonstrated in 7% of tested cattle (6 of 85, CI: 2.6-14.7%). Concerning these animals (No. 4–9: Table 1), haematocrit values were within the normal range in the case of four cattle co-infected with *A. marginale/A. ovis* (No. 5, 7–9) but were slightly lower in animal No. 4 harboring only *B. major*. In addition, cow No. 6 infected with *T. buffeli* (but not with *B. major* or *A. marginale/A. ovis*) was anaemic (Table 1). *B. major* and *T. buffeli* detected in this study had the highest (99%) sequence homology (with one nucleotide difference) to a French (GenBank: GU194290) and Spanish isolate (GenBank: DQ287959), respectively.

*A. marginale/A. ovis msp4* PCR positivity was detected in 20 cattle (prevalence: 23.5%, CI: 15-34%), including four asymptomatic cows with *Babesia*/*Theileria* infection (Table 1). Sequencing was successful from two of the latter animals, revealing *A. marginale* with a sequence identity closest (95%) to *A. ovis. A. phagocytophilum msp2* PCR positivity was shown in 11 animals (prevalence 13%, CI: 6.6-22%) that were all PCR-negative for piroplasms. Four cattle had concurrent *A. phagocytophilum* and *A. marginale*/*A. ovis* infection. During the study no clinical cases attributable to *Anaplasma* spp. were noted in the PCR-positive animals.

### Discussion

Infection of the first animal in this study, which was grazing the same pastures for two years, may indicate that *B. major* has already been present in northeastern Hungary for some time. Alternatively, taking into account that *B. major* was hitherto not detected, it may have been recently introduced. In a region with endemic stability, the majority of locally born calves are exposed to babesiae while protected by maternal immunity and consequently develop long-lasting immunity to subsequent, homologous infection [1]. Therefore, clinical manifestations in the study herd, which was transferred to northeastern Hungary 1.5 months prior to the disease outbreak, may be explained by the absence of this innate resistance. Because the incubation period of bovine babesiosis is usually 1–3 weeks [1], all affected animals might have acquired their infection in northeastern Hungary. In support of this, the tick species *H. punctata* (which is the vector of both *B. major* and *T. buffeli* [23, 24] and is known to occur in Hungary [25]) was found in the pasture of affected animals during the present study (data not shown).

Bovine babesiosis caused by *B. divergens* was reported in Hungary from 1958 to 2005 and showed a steady decline [3]. In the country, *I. ricinus* (the vector of *B. divergens*) has its peak adult activity in May [25]. Accordingly, bovine babesiosis caused by *B. divergens* produced infections in the early summer [3]. On the contrary, in this study relevant clinical signs were noted in November, December and January. Thus, results of the present study might extend the seasonality of bovine babesiosis in Hungary, i.e., it can affect permanently pastured cattle during mild winters, which allow tick activity.

To the best of our knowledge, this is the first evidence regarding the occurrence of *B. major* and *T. buffeli* in central and eastern Europe. In southwestern Europe, where *T. buffeli* has been present for a long time, it usually has a high infection rate in cattle [9, 13]. This may reflect endemic stability in relevant countries [2] and would partly explain why this piroplasm does not appear to affect cattle [9, 13]. However, with the emergence of *T. buffeli* in formerly non-endemic countries, such as Hungary, even this mildly pathogenic species may be involved in pathologies, as shown here for the first time in central and eastern Europe. A similar phenomenon was observed during the emergence of *T. buffeli* in southern Italy between 1954 and 1995 [10] and more recently in the southern hemisphere for relevant genotypes of the *T. orientalis* complex [16].

Interestingly, a high proportion of animals were infected with *A. phagocytophilum* (also reported here for the first time in Hungarian cattle). However, during this study *A. phagocytophilum* was not detected in *Babesia*- or *Theileria*-infected animals, and therefore it most likely did not contribute to the anaemia of cattle affected by piroplasmosis.

The contribution of *A. marginale*/*A. ovis* to the clinical manifestations can also be discounted, because all three animals with lower haematocrit values were anaplasma-free. It was reported that a *T. buffeli*-carrier state in cattle may confer increased resistance to *A. marginale* infection, most likely involving non-specific, cell-mediated immunity [26]. Here, all three cattle that had concurrent *Anaplasma* and *Theileria* infections were healthy, unlike another animal, which was infected only with *T. buffeli*. Based on these results, further studies are warranted regarding whether the presence of these two pathogens is mutually protective.

### Conclusions

The above findings extend the geographical range of *B. major* and *T. buffeli* in Europe. Although both piroplasm species are thought to cause mostly benign infections, in the present study *B. major* may have been responsible for the deaths of two cattle in the herd and *T. buffeli* for anaemia in another animal. *A. phagocytophilum* was detected for the first time in Hungarian cattle with relatively high prevalence (i.e., epidemiological importance) but no associated signs (i.e., low clinical importance).
